# Regulatory Variants in the KRAS 3′UTR and Intron 2 Are Associated with Breast Cancer Susceptibility Through Independent and Combinatorial Effects in a Mexican Population

**DOI:** 10.3390/biomedicines14040948

**Published:** 2026-04-21

**Authors:** Asbiel Felipe Garibaldi-Ríos, Luis E. Figuera, Belinda Claudia Gómez-Meda, Guillermo Moisés Zúñiga-González, Ingrid Patricia Dávalos-Rodríguez, Patricia Montserrat García-Verdín, Ana María Puebla-Pérez, Irving Alejandro Carrillo-Dávila, Martha Patricia Gallegos-Arreola

**Affiliations:** 1 División de Genética, Centro de Investigación Biomédica de Occidente (CIBO), Centro Médico Nacional de Occidente (CMNO), Instituto Mexicano del Seguro Social (IMSS), Guadalajara 44340, Jalisco, Mexico; asbiel.garibaldi4757@alumnos.udg.mx (A.F.G.-R.); luisfiguera@yahoo.com (L.E.F.); patricia.garcia@alumnos.udg.mx (P.M.G.-V.);; 2 Doctorado en Genética Humana, Centro Universitario de Ciencias de la Salud (CUCS), Universidad de Guadalajara (UdeG), Guadalajara 44340, Jalisco, Mexico; 3 Instituto de Genética Humana “Dr. Enrique Corona Rivera”, Departamento de Biología Molecular y Genómica, Centro Universitario de Ciencias de la Salud (CUCS), Universidad de Guadalajara (UdeG), Guadalajara 44340, Jalisco, Mexico; belinda.gomez@academicos.udg.mx; 4 División de Medicina Molecular, Centro de Investigación Biomédica de Occidente (CIBO), Centro Médico Nacional de Occidente (CMNO), Instituto Mexicano del Seguro Social (IMSS), Sierra Mojada 800, Col. Independencia, Guadalajara 44340, Jalisco, Mexico; 5 Laboratorio de Inmunofarmacología, Centro Universitario de Ciencias Exactas e Ingenierías, Universidad de Guadalajara (UdeG), Guadalajara 44430, Jalisco, Mexico

**Keywords:** *KRAS*, regulatory variants, 3′UTR, intronic enhancer, breast cancer susceptibility, microRNA binding, multilocus analysis, eQTL, Mexican population, non-coding variants

## Abstract

**Background:** Breast cancer (BC) is a leading cause of cancer-related mortality worldwide and a major public health concern in Mexico. Regulatory variants in *KRAS*, particularly within the 3′UTR and intronic regions, may influence gene expression through microRNA binding and transcriptional regulation. **Methods:** Five regulatory single-nucleotide variants (SNVs) in *KRAS* (rs12228277, rs1137196, rs8720, rs12587, and rs12245) were genotyped in BC patients and cancer-free controls. Associations were evaluated using odds ratios (ORs) with 95% confidence intervals (CIs), adjusting for age, alcohol, and tobacco use. Multiple testing was corrected using the Benjamini–Hochberg false discovery rate (FDR). Linkage disequilibrium (LD), multilocus combinations, and in silico functional analyses were also performed. **Results:** Variants rs12228277, rs1137196, rs8720, and rs12245 showed significant genotype-level associations with BC susceptibility, all remaining significant after FDR correction (pFDR < 0.05). No clinicopathological associations remained significant after correction in single-variant analyses. Multilocus analysis identified specific high-risk combinations (e.g., involving rs12228277, rs1137196, and rs8720) associated with increased BC susceptibility. At the nominal level, these combinations showed associations with clinicopathological features, including hormone receptor–positive status (PR and ER), proliferation markers, and Luminal B subtype; however, none remained significant after FDR correction. LD analysis indicated weak linkage among variants. In silico analyses suggested potential regulatory effects on microRNA binding and *KRAS* expression. **Conclusions:** Regulatory variants in *KRAS* are associated with BC susceptibility through independent effects and potential combinatorial patterns. These findings support the relevance of non-coding variation in cancer risk and warrant further functional and replication studies.

## 1. Introduction

Breast cancer (BC) remains the most frequently diagnosed malignancy among women worldwide and represents a major cause of cancer-related mortality [1]. In Mexico, BC is the leading cancer in women and continues to pose a significant public health challenge [2,3]. Despite improvements in screening and treatment strategies, the disease exhibits marked biological and clinical heterogeneity, suggesting that additional genetic and regulatory factors may contribute to individual susceptibility and tumor behavior [4,5,6,7,8,9].

Breast carcinogenesis is a multistep process driven by the accumulation of genetic and epigenetic alterations that disrupt key signaling pathways involved in cell proliferation, survival, and differentiation [10,11]. Among the genes implicated in these pathways, *KRAS* encodes a small GTPase that functions as a molecular switch downstream of receptor tyrosine kinases, regulating MAPK and PI3K signaling cascades [12,13]. Although activating somatic mutations in *KRAS* are less frequent in BC compared to other malignancies [14,15], dysregulation of *KRAS* expression has been associated with aggressive phenotypes and poor clinical outcomes [16,17].

Beyond coding mutations, increasing evidence suggests that non-coding regulatory variants may influence cancer susceptibility through modulation of gene expression [18,19,20,21]. The 3′ untranslated region (3′UTR) of *KRAS* is highly conserved and serves as a critical regulatory platform for microRNA (miRNA) binding [22,23]. Variants within this region may alter miRNA–mRNA interactions, potentially affecting transcript stability or translational efficiency [24,25]. Additionally, intronic variants may contribute to transcriptional regulation through enhancer activity, alternative splicing modulation, or intron-mediated enhancement mechanisms [26,27].

Several *KRAS* 3′UTR variants have been evaluated in different types of cancer [28,29,30,31]; however, data regarding other potentially regulatory variants, particularly within intronic regions, remain limited. Moreover, the combined effect of multiple regulatory variants on BC susceptibility and clinicopathological characteristics has not been thoroughly explored, especially in Latin American populations, where genetic background and allele frequencies may differ from those reported in European or Asian cohorts.

In this context, the present study aimed to evaluate the association of five *KRAS* regulatory variants located in intron 2 and the 3′UTR with BC risk and clinicopathological features in Mexican women. These five variants were selected based on three criteria: (i) their location within functionally relevant regulatory regions of *KRAS* (the 3′UTR and intron 2), which are known to harbor miRNA binding sites and transcriptional regulatory elements; (ii) prior evidence of association with cancer susceptibility or *KRAS* expression in other tumor types, including colorectal and lung cancer [29,32,33]; and (iii) their representation of distinct regulatory mechanisms (post-transcriptional regulation via miRNA binding for 3′UTR variants, and transcriptional enhancer activity for the intronic variant rs12228277). These selection criteria were applied to prioritize variants with the highest prior biological plausibility for influencing *KRAS* regulation in a cancer context. Additionally, in silico analyses were performed to explore their potential functional relevance at the post-transcriptional and regulatory levels.

We hypothesize that regulatory variants in *KRAS* independently contribute to BC susceptibility and may exhibit combinatorial effects influencing clinicopathological features.

## 2. Materials and Methods

### 2.1. Study Design and Population

A total of 650 women with histopathologically confirmed BC were enrolled across two recruitment periods corresponding to institutional ethics protocol R-2019-1305-037, and 492 cancer-free controls were included in the study. All participants were unrelated Mexican women over 18 years of age and reported Mexican ancestry for at least two generations. Controls had no personal history of cancer. Individuals who had received blood transfusions within two months prior to sampling were excluded.

The minimum required sample size per group was estimated a priori using the infinite-populations formula n = Zα^2^pq/d^2^ [34], with Zα = 1.96 (95% confidence), d = 0.05 (margin of error), and the minor allele frequency (MAF) per variant in the Mexican population (MXL), as reported in the Ensembl database (GRCh38.p13) [35]. The calculated minimums were: rs12228277 (A; MAF = 0.203) = 124 samples; rs1137196 (T; MAF = 0.492) = 192 samples; rs8720 (T; MAF = 0.445) = 190 samples; rs12587 (T; MAF = 0.445) = 190 samples; rs12245 (A; MAF = 0.445) = 190 samples. The most conservative estimate (rs1137196, *n* = 192) was used to define the target recruitment. All analyzed SNVs met the minimum required sample size in both the BC and control groups, indicating that the sample size was sufficient to detect moderate to large effect sizes based on allele frequency estimates.

Clinical and epidemiological information was obtained from medical records at the División de Genética, Centro de Investigación Biomédica de Occidente (CIBO-IMSS), Mexico. Variables analyzed in BC patients included tumor stage, histological type, molecular subtype, lymph node status, Ki-67 index, presence of metastasis, response to chemotherapy, and treatment-related toxicity. Additional demographic and reproductive variables included age at diagnosis, age at menarche and menopause, number of pregnancies, breastfeeding duration, body mass index (BMI), alcohol consumption, and smoking status.

The study was conducted in accordance with the Declaration of Helsinki and approved by the institutional ethics committee of CIBO-IMSS under protocol R-2019-1305-037. All participants had previously provided written informed consent for genetic analysis and future research use of their samples. All analyses were performed anonymously.

### 2.2. DNA Extraction and Genotyping

DNA was extracted from peripheral blood leukocytes using a standard protocol. DNA concentration and purity were determined by spectrophotometry using a NanoDrop 2000 (Thermo Fisher Scientific, Waltham, MA, USA). Purity was considered optimal with an A260/A280 ratio between 1.8 and 1.9, and an A260/A230 ratio between 1.8 and 2.2. All samples were normalized to a working concentration range of 20–50 ng/µL before genotyping.

Genotyping of the five KRAS variants was performed using TaqMan allelic discrimination assays (Thermo Fisher Scientific) by real-time PCR on a CFX96 Real-Time PCR Detection System (Bio-Rad Laboratories, Hercules, CA, USA). The specific commercial assays used were: rs12228277 (C__31385938_10), rs1137196 (C_175867240_10), rs8720 (C_189578752_10), rs12587 (C__12104199_10), and rs12245 (C_176064436_10). Each reaction was performed in a total volume of 15 µL, consisting of 6.25 µL of 2X TaqMan Genotyping Master Mix, 0.32 µL of the 40X SNP Genotyping Assay, 3.43 µL of ultrapure water, and 5.0 µL of genomic DNA (100–250 ng total). The thermal cycling conditions consisted of an initial activation at 95 °C for 10 min, followed by 40 cycles of denaturation at 95 °C for 15 s and annealing/extension at 60 °C for 1 min. Automated genotype calling was performed using the system’s integrated software based on the fluorescence signals of the VIC and FAM-labeled probes. For quality control, 10% of the samples were randomly re-genotyped with 100% concordance.

### 2.3. In Silico Functional Analysis

To explore the potential biological impact of the analyzed variants, in silico analyses were performed using publicly available databases.

Variants located in the 3′UTR (rs1137196, rs8720, rs12587, and rs12245) were evaluated for possible alterations in microRNA binding sites using miRNASNP v3 [36] (http://bioinfo.life.hust.edu.cn/miRNASNP/, accessed on 20 January 2026) and PolymiRTS v3.0 [37] (http://compbio.uthsc.edu/miRSNP/, accessed on 25 January 2026).

The intronic variant rs12228277 was analyzed using RegulomeDB [38] (https://regulomedb.org/regulome-search/, accessed on 25 January 2026) and HaploReg v4.1 [39] (https://pubs.broadinstitute.org/mammals/haploreg/haploreg.php, accessed on 25 January 2026) to assess regulatory potential, including transcription factor binding, DNase hypersensitivity sites, chromatin states, and enhancer annotations.

Additionally, expression quantitative trait loci (eQTL) analyses were conducted using the GTEx database [40] (https://gtexportal.org/home/, accessed on 2 February 2026) to determine whether these variants are associated with gene expression changes in breast tissue, providing further insight into their potential regulatory effects in a tissue-relevant context.

### 2.4. Statistical Analysis

Allele and genotype frequencies were calculated by direct counting. Hardy–Weinberg equilibrium (HWE) was assessed exclusively in the control group using the chi-square test with one degree of freedom, as is methodologically appropriate for case–control genetic association studies.

Associations between genetic variants and BC risk were evaluated by calculating odds ratios (ORs) and 95% confidence intervals (CIs) under codominant, dominant, and recessive inheritance models. Logistic regression analyses for susceptibility were adjusted for age, tobacco use, and alcohol consumption, as these variables differed significantly between cases and controls. The Benjamini–Hochberg false discovery rate (FDR) method was applied globally across all susceptibility comparisons. Associations with an FDR-adjusted *p*-value (pFDR) < 0.05 were considered statistically significant after correction for multiple comparisons.

Associations between genotypes and clinicopathological characteristics were assessed using chi-square or Fisher’s exact tests. For clinicopathological analyses, FDR correction was applied within each single nucleotide variant (SNV) due to the exploratory nature and reduced sample size of subgroup comparisons; therefore, these results are not directly comparable to susceptibility analyses. Linkage disequilibrium (LD) parameters (D′ and r^2^) were estimated using SHEsisPlus software [41].

Multilocus genotype combinations were generated by concatenating genotypes across loci. Frequency distributions were calculated using RStudio V. 2025.05.0+496 (tidyverse and readxl packages), and associations were evaluated using logistic regression with the epitools package. For multilocus susceptibility analyses, FDR correction was applied globally across all pairwise-combination comparisons. Multilocus analyses were not adjusted for additional covariates due to reduced sample size within genotype combinations; therefore, the estimated effects should be interpreted cautiously.

Normality of continuous variables was assessed using the Kolmogorov–Smirnov test to determine the use of parametric (Student’s *t*-test) or non-parametric (Mann–Whitney U test) comparisons. A *p*-value < 0.05 was considered statistically significant.

The number of samples included in each analysis varied across SNVs due to reagent availability during genotyping; however, all analyzed SNVs met or exceeded the pre-specified minimum sample size in both groups, ensuring adequate statistical power for all comparisons.

## 3. Results

### 3.1. Clinical and Epidemiological Characteristics

A total of 650 women with BC and 492 cancer-free controls were included in the final analytical dataset.

The mean age at diagnosis in BC patients was 53.26 ± 11.31 years, compared with 53.84 ± 11.64 years in controls, showing no statistically significant difference (*p* = 0.412) (Table 1). Within the BC cohort, most patients were postmenopausal (64%), overweight or obese (70%), and diagnosed with ductal carcinoma (91%). Advanced clinical stages (III–IV) were observed in 57% of cases, and 23% presented metastasis. A Ki-67 index ≥ 20% was detected in 59% of tumors. Regarding treatment response, 43% achieved a complete response to chemotherapy (Table 1). Alcohol and tobacco consumption differed significantly between cases and controls (*p* = 0.0001 and *p* = 0.001, respectively).

### 3.2. Individual Association of KRAS Variants with BC Risk

All effective sample sizes exceeded the pre-specified minimum of 190 participants per group. Genotype distributions in the control group were consistent with HWE for all analyzed variants (rs12228277: χ^2^ = 0.007, *p* = 0.933; rs1137196: χ^2^ = 0.027, *p* = 0.871; rs8720: χ^2^ = 0.046, *p* = 0.831; rs12587: χ^2^ = 0.081, *p* = 0.777; rs12245: χ^2^ = 0.203, *p* = 0.653). HWE was assessed exclusively in the control group, as recommended for case–control genetic association studies.

As shown in Table 2, comparisons of allele and genotype frequencies between groups revealed several statistically significant associations. All nominally significant associations (*p* < 0.05) were further evaluated using the Benjamini–Hochberg FDR correction applied to the 25 tests performed (five SNVs across five genetic models). Most associations remained significant after FDR adjustment (pFDR < 0.05), supporting the robustness of the observed signals. The only association that did not remain significant after correction was the dominant model for rs8720 (*CT* + *CC* vs. *TT*; *p* = 0.0588; pFDR = 0.0918). FDR-adjusted *p*-values are reported in Table 2.

For rs12228277, the *A* allele was more frequent among BC patients than in controls and was associated with increased risk (OR = 1.99; 95% CI: 1.66–2.38; *p* < 0.0001; pFDR < 0.0001). Consistently, both the *AT* genotype (OR = 2.00; 95% CI: 1.54–2.59; pFDR < 0.0001) and the AA genotype (OR = 3.91; 95% CI: 2.57–5.95; pFDR < 0.0001) were associated with increased risk. Significant associations were also observed under the dominant (OR = 2.30; 95% CI: 1.80–2.94; pFDR < 0.0001) and recessive models (OR = 2.75; 95% CI: 1.85–4.09; pFDR < 0.0001).

For rs1137196, the *GG* genotype was associated with increased BC risk under both the codominant model (OR = 2.31; 95% CI: 1.61–3.33; pFDR < 0.0001) and the recessive model (OR = 2.77; 95% CI: 2.01–3.82; pFDR < 0.0001). In addition, the *G* allele was associated with increased risk (OR = 1.56; 95% CI: 1.28–1.88; pFDR < 0.0001). The heterozygous *GT* genotype showed a borderline association that did not remain significant after FDR correction (pFDR = 0.0917).

For rs8720, the *C* allele (OR = 1.54; 95% CI: 1.27–1.87; pFDR < 0.0001) and the *CC* genotype (OR = 2.14; 95% CI: 1.48–3.09; pFDR = 0.0001) were associated with increased BC risk. Similar results were observed under the recessive model (OR = 2.12; 95% CI: 1.58–2.85; pFDR < 0.0001). No significant association was observed for the heterozygous genotype or the dominant model after FDR correction.

For rs12587, no significant differences in allele or genotype frequencies were observed between cases and controls under any genetic model (pFDR > 0.05).

For rs12245, the *AT* genotype was associated with decreased risk (OR = 0.55; 95% CI: 0.37–0.82; pFDR = 0.0065). However, no significant associations were observed for allele frequencies or other genetic models after FDR correction.

### 3.3. Association with Clinicopathological Variables

When genotypic distributions were compared with clinicopathological characteristics, multiple nominal associations (*p* < 0.05) were identified across the analyzed variants. However, none of these associations remained statistically significant after Benjamini–Hochberg FDR correction (pFDR > 0.05), indicating a lack of robust evidence supporting an association between these SNVs and clinical variables in this dataset. Therefore, all findings from this analysis should be considered exploratory (Table 3).

At the nominal level, several suggestive associations were observed. For rs12587, the *GT* genotype showed a positive association with PR-positive status (OR = 1.66, 95% CI: 1.08–2.55, *p* = 0.0218) and an inverse association with high Ki-67 levels (OR = 0.53, 95% CI: 0.32–0.87, *p* = 0.0126).

For rs12228277, the *AT* genotype was nominally associated with a lower likelihood of lymph node metastasis (OR = 0.68, 95% CI: 0.46–0.99, *p* = 0.0419) and the Luminal B subtype (OR = 0.65, 95% CI: 0.45–0.93, *p* = 0.0187). In addition, both the *AT* and *AA* genotypes showed inverse nominal associations with PR-positive status (*AT*: OR = 0.60, 95% CI: 0.41–0.88, *p* = 0.0090; *AA*: OR = 0.54, 95% CI: 0.33–0.87, *p* = 0.0118).

For rs12245, the *AT* genotype showed a nominal association with PR-positive status (OR = 1.88, 95% CI: 1.12–3.17, *p* = 0.0174). Similarly, for rs1137196, the *GG* genotype was nominally associated with the Luminal B subtype (OR = 1.64, 95% CI: 1.03–2.63, *p* = 0.0400).

### 3.4. Linkage Disequilibrium and Multilocus Analysis

Linkage disequilibrium (LD) analysis among rs12228277, rs1137196, rs8720, rs12587, and rs12245 yielded the following D’ values: rs12228277–rs1137196 (0.14), rs12228277–rs8720 (0.29), rs12228277–rs12587 (0.35), rs12228277–rs12245 (0.32), rs1137196–rs8720 (0.29), rs1137196–rs12587 (0.30), rs1137196–rs12245 (0.31), rs8720–rs12587 (0.28), rs8720–rs12245 (0.22), and rs12587–rs12245 (0.25). Furthermore, r2 values were notably low: rs12228277–rs1137196 (0.01), rs12228277–rs8720 (0.08), rs12228277–rs12587 (0.11), rs12228277–rs12245 (0.10), rs1137196–rs8720 (0.03), rs1137196–rs12587 (0.08), rs1137196–rs12245 (0.07), rs8720–rs12587 (0.03), rs8720–rs12245 (0.04), and rs12587–rs12245 (0.04). Global analysis showed a χ^2^ value of 48.201 (*p* < 0.0001). These results indicate a weak linkage disequilibrium among the variants, justifying the individual and combined association analyses. Consequently, a pairwise combination approach was implemented to provide an exploratory assessment of combined genotype effects on risk magnitude and direction (Table 4).

### 3.5. Pairwise Genotypic Combinations and BC Risk

The significant pairwise genotypic combinations associated with BC risk are summarized in Table 4. Among these, the strongest association was observed for rs12228277_rs1137196 (*AT_GG*; OR = 7.48, 95% CI: 3.72–15.07; pFDR < 0.0001), followed by rs1137196_rs8720 (*GG_CC*; OR = 4.79, 95% CI: 2.34–9.80; pFDR = 0.0006. Additional significant risk associations included rs12228277_rs8720 (*AT_CC*; OR = 3.02, 95% CI: 1.60–5.69; pFDR = 0.0106), rs8720_rs12587 (*CC_GT*; OR = 2.98, 95% CI: 1.61–5.52; pFDR = 0.0106), rs12228277_rs12587 (*AT_GG*; OR = 2.86, 95% CI: 1.53–5.33; pFDR = 0.0151; *AT_TT*: OR = 2.72, 95% CI: 1.46–5.06; pFDR = 0.0213; *AA_GT*: OR = 3.11, 95% CI: 1.49–6.50; pFDR = 0.0275), rs12228277_rs8720 (AA_TT; OR = 4.20, 95% CI: 1.62–10.88; pFDR = 0.0356), and rs12228277_rs1137196 (*AA_GT*; OR = 2.99, 95% CI: 1.37–6.52; pFDR = 0.0434).

Notably, the directionality of risk associations was not uniformly determined by the total number of accumulated alternative alleles. Most combinations carrying three or four alternate alleles showed consistently elevated ORs. However, the combination *AT_TT* for rs12228277_rs12587, which carries only one alternate allele (the A allele of rs12228277 in heterozygosity), was also significantly associated with increased risk (OR = 2.72; pFDR = 0.0213). Similarly, *AA_TT* for rs12228277_rs8720, carrying two alternate alleles exclusively at rs12228277, showed the third highest OR among all significant combinations (OR = 4.20; pFDR = 0.0356). These observations suggest that the effect of rs12228277 alternate alleles on BC risk may be partially independent of co-occurring variants at other loci.

The sole protective association surviving FDR correction was rs1137196_rs12245 (*TT_AT*; OR = 0.37, 95% CI: 0.19–0.74; pFDR = 0.0434), in which the alternate allele is carried exclusively at rs12245 in heterozygosity. This finding is consistent with the individually protective effect of the *AT* genotype of rs12245 observed in single-variant analysis (OR = 0.55; pFDR = 0.0065), suggesting that the protective signal at this locus is maintained in the multilocus context. Some estimates showed wide confidence intervals, reflecting reduced sample sizes within specific genotype strata, and should be interpreted accordingly.

### 3.6. Association Between Genotypic Combinations and Clinical–Pathological Profile

Multilocus combinations showed several associations with clinicopathological variables at the nominal level (*p* < 0.05). However, none of these associations remained statistically significant after false discovery rate correction (all pFDR > 0.05) (Table 5). Therefore, these findings should be interpreted cautiously as exploratory signals rather than conclusive associations.

At the nominal level, associations were observed across hormone receptor status, proliferation markers, and tumor progression features. The *GT_AT* combination of the rs12587_rs12245 pair was associated with increased PR positivity (OR = 5.01, 95% CI: 1.96–12.75, *p* = 0.0008). Similarly, rs12228277_rs12587 (*TT_GT*) (OR = 3.73, 95% CI: 1.40–9.98, *p* = 0.0114) and rs1137196_rs12587 (*GT_GT*) (OR = 2.58, 95% CI: 1.12–5.92, *p* = 0.0359) also showed associations with higher PR positivity.

In contrast, proliferation-related features showed an opposite pattern. The *GT_AT* combination in rs12587_rs12245 was associated with a reduced likelihood of high Ki-67 levels (OR = 0.23, 95% CI: 0.09–0.60, *p* = 0.0030), and similarly, rs1137196_rs12587 (*GT_GT*) was associated with a lower Ki-67 index (OR = 0.34, 95% CI: 0.14–0.84, *p* = 0.0220).

Additionally, tumor progression and subtype-related features were identified. Lymph node metastasis was associated with the *TT_AT* combination in rs12228277_rs12245 (OR = 4.36, 95% CI: 1.70–11.21, *p* = 0.0036), while ER positivity was associated with the *AT_TT* configuration in rs12228277_rs1137196 (OR = 2.51, 95% CI: 1.12–5.64, *p* = 0.0307). Regarding intrinsic subtype, the *AT_AA* (OR = 0.32, 95% CI: 0.12–0.83, *p* = 0.0318) and *AT_TT* (OR = 0.38, 95% CI: 0.15–0.95, *p* = 0.0418) combinations in rs12228277_rs12245 were associated with a decreased likelihood of Luminal B subtype.

### 3.7. In Silico Analysis of the Variants

#### 3.7.1. miRNAs Targeting the Genomic Regions of the SNVs Under Study

**Figure 1:**
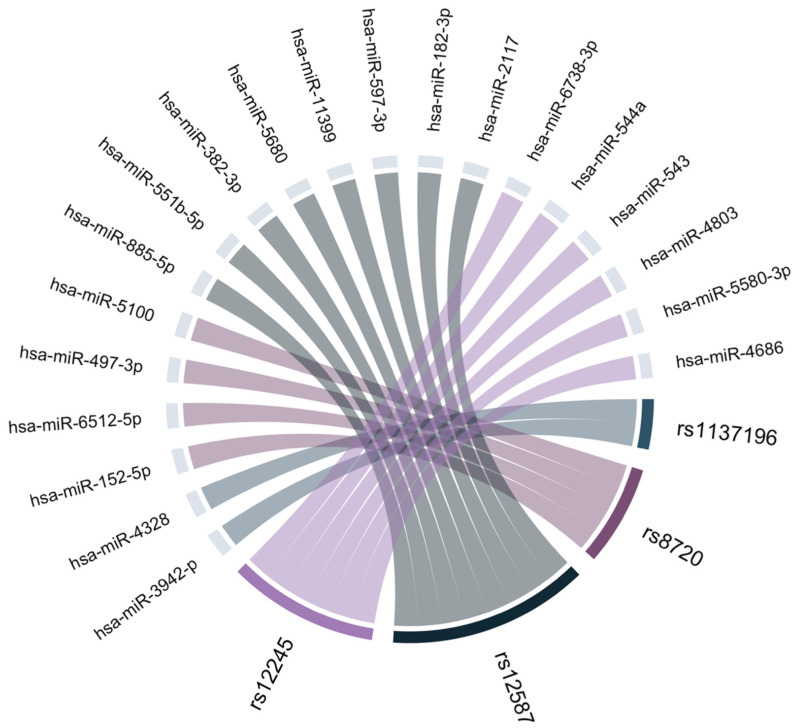
miRNAs targeting the genomic regions of the studied SNVs.

Using the tools miRNA SNP V3 (http://bioinfo.life.hust.edu.cn/miRNASNP; accessed 20 January 2026) and PolymiRTS Database 3.0 (https://compbio.uthsc.edu/miRSNP/home.php; accessed 25 January 2026), it was predicted that the variants located in the 3′UTR region of *KRAS* (rs1137196, rs8720, rs12587, and rs12245) overlap with binding sites of several miRNAs that could potentially regulate *KRAS* expression (Figure 1).

#### 3.7.2. Regulatory Analysis of the rs12228277 Variant

In the in silico functional analysis of the rs12228277 variant, RegulomeDB [38] assigned a score of 1f, indicating experimental evidence of regulatory elements in the evaluated region (Table 6). A total of 82 DNase I hypersensitivity peaks were identified, suggesting high chromatin accessibility. Additionally, binding of the transcription factor ATF2 was detected at this site, supported by ChIP-seq data.

The chromatin states associated with this variant included Active enhancer 2, Weak enhancer, Genic enhancer 1, Strong transcription, and Weak transcription, suggesting regulatory activity within this intronic region. Analysis using HaploReg v4.1 showed that rs12228277 is located in regions enriched for enhancer marks and DNase sites across multiple tissues, particularly blood (BLD), lung (LNG), and vascular tissue (VAS). Furthermore, this variant was found to alter binding motifs for transcription factors such as NF-κB and Mrg1::Hoxa9.

#### 3.7.3. Variant-Regulated Expression Analysis (eQTL) in Breast Tissue

In the eQTL analysis for *KRAS* in breast tissue (Table 7), the rs12228277 variant showed no association with gene expression (*p* = 0.92; NES = 0.0038), indicating no regulatory effect in this tissue. In contrast, the variants rs8720, rs12587, and rs12245 showed significant associations with *KRAS* expression, all with *p*-values below 0.001 and negative NES values (–0.094 for rs8720 and rs12245; –0.093 for rs12587), indicating decreased expression associated with the alternate allele. Finally, no data were available for the rs1137196 variant in the platform, and therefore no information could be obtained for this locus.

### 3.8. Integrative Model of Independent and Allele-Specific Effects of KRAS Regulatory Variants in BC Susceptibility

**Figure 2:**
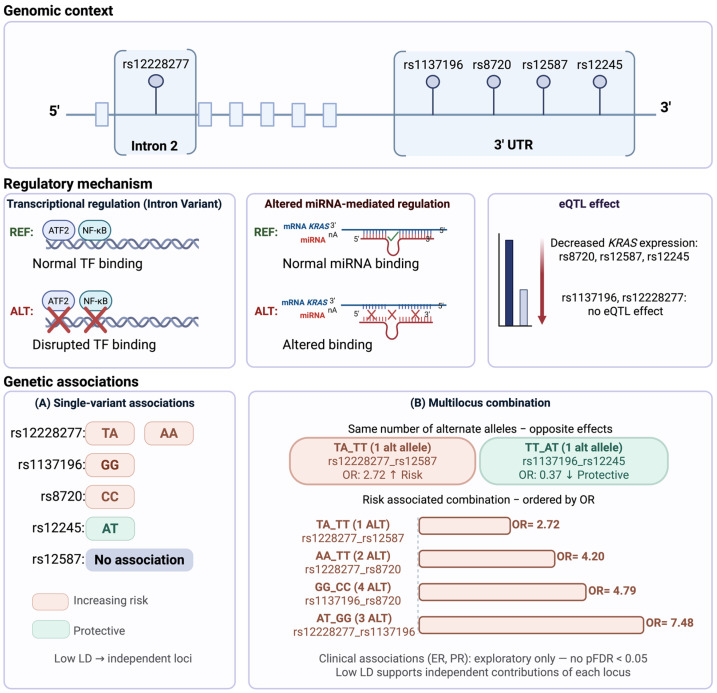
Schematic representation of the genomic context, functional mechanisms, and genetic effects of the studied variants. The upper panels contrast in silico predictions of transcriptional regulation (rs12228277, intron 2) and miRNA-mediated post-transcriptional regulation (3′UTR variants) with eQTL expression data in normal breast tissue, which confirmed expression modulation only for rs8720, rs12587, and rs12245. At the genetic level, single-variant analyses (A) reveal allele-specific risk and protective profiles. Multilocus combinations (B) demonstrate that effect directionality is determined by the identity of the alternate allele rather than by total allele accumulation: combinations carrying the rs12228277-*A* allele consistently confer increased risk, while the rs12245-*AT* genotype maintains its protective effect in the multilocus context. Clinical associations did not survive FDR correction and should be interpreted as exploratory. Created in BioRender. Garibaldi, A. (2026). https://BioRender.com/v948van (accessed 12 April 2026).

To integrate the genetic association findings with the predicted functional mechanisms, we developed a proposed model summarizing the role of *KRAS* regulatory variants in BC susceptibility (Figure 2). This model illustrates that individual variants exhibit genotype-specific effects, with risk and protective associations driven by particular allelic configurations rather than by a simple gradient of alternate allele accumulation. The low linkage disequilibrium observed among all five loci supports their independent contribution to disease susceptibility, justifying both individual and combinatorial analyses.

At the functional level, in silico annotations suggest distinct regulatory mechanisms depending on genomic location. The intronic variant rs12228277 shows evidence of enhancer-mediated transcriptional regulation, including disruption of ATF2 and NF-κB transcription factor binding motifs, while the 3′UTR variants are predicted to alter miRNA-mediated post-transcriptional regulation. Consistent with this distinction, eQTL data in normal breast tissue supported expression modulation for rs8720, rs12587, and rs12245, but not for rs12228277 or rs1137196, pointing toward tissue-specific and mechanism-specific regulatory roles.

Multilocus analysis identified nine risk-associated and one protective combination surviving FDR correction. Notably, the directionality of multilocus effects was allele-specific: the A allele of rs12228277 was present in seven of nine significant risk combinations and conferred increased risk even as a single alternate allele, while the *AT* genotype of rs12245 maintained its protective signal in the multilocus context. No associations between genotypic combinations and clinicopathological features survived FDR correction; nominally observed associations with PR status were in the direction of PR-positive enrichment and should be considered strictly exploratory.

## 4. Discussion

BC remains one of the leading causes of cancer-related mortality among women worldwide and represents a major public health concern in Mexico [1,2]. In this study, we evaluated the association of five regulatory variants in *KRAS* (rs12228277, rs1137196, rs8720, rs12587, and rs12245) with BC susceptibility and clinicopathological characteristics in Mexican women. Overall, our results indicate that regulatory variants within *KRAS* are associated with disease susceptibility and may influence tumor-related features.

At the genetic level, four variants showed consistent associations with BC susceptibility after FDR correction. The intronic variant rs12228277 exhibited the strongest and most consistent effect across all genetic models, suggesting a consistent contribution to disease risk. In addition, rs1137196, rs8720, and rs12245, located in the 3′UTR, were also significantly associated with susceptibility, showing genotype-specific risk and protective patterns. In contrast, rs12587 did not show an association with susceptibility.

Although these specific variants have been less extensively studied in BC, previous studies have demonstrated that regulatory variants in *KRAS* are associated with cancer susceptibility in other tumor types [29,33,42,43,44,45]. In particular, variants located in the 3′UTR have been linked to colorectal [46,47] and lung cancer risk [48,49] through their effects on microRNA binding [50,51]. A well-characterized example is the LCS6 variant (rs61764370), which alters the binding of the let-7 microRNA family and has been associated with cancer risk and clinical outcomes [52]. These findings support the biological relevance of post-transcriptional regulation of *KRAS* and are consistent with the associations observed in the present study.

To our knowledge, this is the first study to associate the rs12228277 variant with BC susceptibility and clinical progression. Although this variant has not been previously evaluated in BC, it was recently identified in a colorectal cancer cohort from Colombia [53], suggesting a potentially broader role in oncogenesis within Latin American populations. Interestingly, our data revealed a complex clinical picture for this variant. While the minor A allele is strongly associated with an increased risk of BC development in a dose-dependent manner (OR = 3.91 for the *AA* genotype), its presence in patients showed nominal inverse associations with less aggressive clinical features, demonstrated by an inverse association with axillary lymph node metastasis, the Luminal B subtype, and PR-positive status. This apparent dual effect may reflect stage-specific roles, where the variant contributes to tumor initiation while modulating pathways involved in tumor progression or differentiation.

This dual behavior may be partially explained by the variant’s regulatory potential [54,55,56,57]. In silico annotations provide strong functional evidence (RegulomeDB score 1f) that rs12228277 resides within an active intronic enhancer. Crucially, HaploReg data indicate that this variant alters the binding motifs for significant transcription factors, including NF-κB and ATF2, which are well-known regulators of cellular proliferation and tumor progression. While the precise molecular mechanism remains to be elucidated, the disruption of these regulatory elements suggests a pathway through which this non-coding variant could differentially influence both tumor initiation and subsequent phenotypic characteristics. Further in vitro functional assays are required to validate the specific impact of rs12228277 on enhancer activity and its downstream transcriptional targets.

Variants located in the 3′UTR (rs1137196, rs8720, rs12587, and rs12245) showed patterns consistent with post-transcriptional regulatory effects. These regions are known to modulate mRNA stability and translation through interactions with microRNAs. In this study, rs1137196 and rs8720 were associated with BC risk, supporting the hypothesis that alterations in microRNA binding may contribute to disease susceptibility.

In this study, specific genotypes and alleles of rs1137196, rs8720, and rs12245 significantly modulated BC risk, supporting the hypothesis that alterations in microRNA binding may fundamentally contribute to disease susceptibility [30,58]. In contrast, rs12587 did not affect overall BC risk but rather influenced the clinical phenotype. A relevant finding from eQTL analysis is that rs8720, rs12587, and rs12245 have been associated with decreased *KRAS* expression in normal breast tissue, despite their linkage to altered oncogenic risk or tumor characteristics. This apparent inverse relationship between reduced *KRAS* expression in normal tissue and increased cancer risk suggests a complex regulatory mechanism, where reduced baseline expression may alter cellular sensitivity to oncogenic signaling or reflect compensatory regulatory dynamics during tumorigenesis. This apparent discrepancy highlights the context-dependent nature of regulatory variants, suggesting that effects observed in normal tissue may not directly translate to tumor biology, where global transcriptional and post-transcriptional networks are extensively reprogrammed. One possible explanation, supported by our in silico network, is that these variants modify microRNA binding sites to enhance interactions with suppressive microRNAs in normal tissue. However, during tumor development, the global regulatory environment undergoes profound changes, including the depletion of specific microRNAs [59,60]. In this oncogenic context, altered binding sites may contribute to a regulatory imbalance rather than a simple reduction in gene expression, uncoupling *KRAS* from normal post-transcriptional control.

This context- and tissue-dependent behavior becomes particularly evident when comparing our genotypic associations with other malignancies across diverse populations. For instance, the *CC* genotype and the *C* allele of rs8720 demonstrated a strong association with BC susceptibility in our cohort. This finding is highly consistent with the risk profiles observed in colorectal cancer within the Chinese [29] and Mexican populations [32], as well as in laryngeal squamous cell carcinoma in Iran [61]. The remarkable similarity of these results across different ethnic groups and tumor types suggests that the post-transcriptional disruption caused by rs8720 represents a broad, cross-tissue mechanism of oncogenic dysregulation.

Conversely, our results for rs1137196 contrast with previous literature. While we found that the *GG* genotype and G allele significantly increased BC risk and were associated with the Luminal B subtype, the heterozygous *TG* genotype showed a protective effect. This pattern may suggest a potential overdominance effect; however, it could also reflect sampling variability or unmeasured confounding, and therefore should be interpreted cautiously. A study in colorectal cancer found no such association in a Chinese population [41]. This discrepancy may be explained by the complex interplay between population genetics and tumor-specific biology. First, underlying genetic ancestry plays a crucial role; differences in minor allele frequencies and genetic backgrounds between the admixed Latin American population in our study and the East Asian cohort likely contribute to these divergent risk profiles. Furthermore, the oncogenic impact of this 3′UTR variant is highly disease-specific. Breast and colorectal cancers are driven by fundamentally distinct signaling pathways and hormonal microenvironments. Consequently, the disruption of specific microRNA regulatory nodes by rs1137196 might be a critical driver for transformation in the breast epithelium, yet remain potentially redundant or inactive in the colon.

A similar pleiotropic behavior was observed for the rs12587 and rs12245 variants. Unlike rs8720, rs12587 did not show a significant association with overall BC susceptibility in our study. This lack of association aligns with negative findings reported for hepatoblastoma in China [62] and colorectal cancer in Mexico [32]. However, it differs from studies linking this variant to an increased risk of Wilms tumor [45], lung cancer [63], and glioma [64] in Asian populations. Interestingly, within our clinical data, the rs12587 *GT* genotype was nominally associated with PR-positive status and a lower proliferative index (Ki-67), suggesting it primarily fine-tunes tumor phenotype rather than initiation.

Finally, rs12245 presented a highly complex, allele-dosage-dependent pattern. In our cohort, the heterozygous AT genotype appeared to confer protection against BC development and correlated nominally with PR-positive tumors, whereas the homozygous TT genotype reversed this effect, acting as a moderate risk factor. This duality reflects the complex behavior of rs12245 reported in the literature; for instance, while Q. H. Liu et al. (2020) [65] correlated it with increased *KRAS* expression in non-Hodgkin lymphoma, Liang et al. (2023) reported a protective association in non-small cell lung cancer [63]. Together, these findings support the hypothesis that some 3′UTR variants may not act as universal, linear drivers of transformation. Instead, they likely fine-tune the clinical phenotype and tumor progression pathways in a highly dynamic manner, reacting to the specific stoichiometry and basal abundance of interacting microRNAs within the local microenvironment.

Furthermore, it is important to emphasize that the current scarcity of studies specifically investigating these *KRAS* regulatory variants in BC cohorts, both in Latin American and international populations, represents a significant challenge for direct comparison within the same oncological context. While we have drawn parallels with other malignancies where these SNVs have been validated, the distinct hormonal and molecular landscapes of BC may influence the regulatory behavior of these variants differently. This gap in the literature underscores the novelty of our findings and highlights the pressing need for further multi-center, population-based studies to determine whether these genetic risk profiles are universal or ethnically specific.

To further dissect the genetic architecture of *KRAS* susceptibility, we evaluated the combinatorial effect of these SNVs. Traditional genetic studies often rely on haplotype analysis to assess multilocus risk; however, our linkage disequilibrium (LD) profiling revealed notably low r-squared values (all < 0.11) among the studied variants. This lack of LD indicates that these loci segregate largely independently, rendering conventional haplotype block construction biologically inappropriate and statistically underpowered for this specific region [66,67]. Consequently, we implemented a pairwise genotypic combination approach to capture potential epistatic interactions, a strategy that, to our knowledge, has not been previously applied to this set of *KRAS* regulatory variants in BC.

This combinatorial analysis suggests potential combinatorial patterns influencing disease susceptibility. While individual variants conferred moderate risk, the presence of multiple alternate alleles was generally associated with increased BC susceptibility, although this effect was not strictly determined by the total number of alternate alleles. The highest risk estimate was observed for the rs12228277_rs1137196 (*AT_GG*; OR = 7.48, 95% CI: 3.72–15.07). However, this result should be interpreted with caution due to the limited number of individuals in specific genotype strata and the width of the confidence intervals. These factors suggest that the magnitude of some effects may be overestimated, although the direction of the association remains generally consistent toward increased risk across most combinations, with few protective configurations. We hypothesize that the observed increase in risk for certain genotype combinations may reflect a potential combined regulatory effect involving both transcriptional and post-transcriptional mechanisms. The intronic variant rs12228277 may influence transcription factor binding and enhancer activity, while 3′UTR variants could alter microRNA-mediated regulation. However, this proposed model remains speculative and requires experimental validation to confirm its biological relevance.

Notably, the effect of multilocus combinations was not strictly dependent on the total number of alternate alleles. Certain combinations carrying a limited number of alternate alleles, particularly those involving rs12228277, still showed strong associations with BC risk. This suggests that the contribution of specific loci may outweigh the cumulative allelic burden in determining risk.

The stratification of our cohort into pairwise genotypic groups inevitably reduced the sample size within specific combinatorial strata. For example, several high-risk combinations were observed in a limited number of individuals within specific genotype strata. This sparse data distribution may inflate the point estimates of ORs and generate wide confidence intervals. Given the low frequency of certain genotype combinations, particularly in multilocus analyses, some effect estimates may be inflated, and these findings should be considered hypothesis-generating rather than definitive. While several associations remained significant after FDR correction, their magnitude should be interpreted with caution. These epistatic models should therefore be viewed as proof-of-concept findings that warrant validation in larger, independent multicenter cohorts.

Importantly, this epistatic burden may contribute to disease susceptibility and may also be associated with clinical characteristics. Unlike our single-variant analyses, which lost statistical significance for clinical parameters after FDR correction, multilocus combinations revealed associations with multiple tumor-related features at the nominal level, although these did not withstand FDR correction and should therefore be considered exploratory findings. The combined polymorphic burden showed heterogeneous associations with clinical features, including markers of more differentiated tumor phenotypes (e.g., increased PR positivity and lower Ki-67 levels) as well as features associated with tumor progression (e.g., lymph node metastasis). Given that none of these associations remained significant after multiple testing correction, these findings should be interpreted as exploratory and hypothesis-generating.

These findings are consistent with a potential biological interplay between regulatory variants. A single regulatory variant might be partially buffered by the tumor microenvironment or compensatory microRNAs [68]. However, a combined regulatory effect may contribute to altered regulation of KRAS-related signaling pathways. We hypothesize that this sustained hyperactivation of the downstream MAPK signaling pathway may contribute to altered proliferative signaling and modulating tumor differentiation and proliferative behavior in a heterogeneous manner [69,70] suggesting that these variants may influence tumor biology in a context-dependent manner, potentially modulating both hormonal signaling and proliferative activity rather than uniformly promoting a more aggressive phenotype. Ultimately, these findings suggest that evaluating *KRAS* regulatory variants as isolated entities may underestimate their oncogenic potential; their combined effects may play a role in susceptibility and potentially influence disease characteristics.

Our study has certain limitations that should be acknowledged. First, although the germline nature of these variants ensures their constitutive presence in the target tissue, the absence of matched tumor biopsies prevented the direct evaluation of their transcriptomic or proteomic impact within the tumor microenvironment. Second, there is a lack of detailed reproductive and hormonal history (such as age at menarche or use of hormone replacement therapy) for the control group. Because this cohort consisted of healthy blood donors, these specific clinical variables were not actively collected for this group during the study. Additionally, while this study focused on an admixed Mexican population, we did not perform a formal quantification of individual genetic ancestry components, such as Native American or European backgrounds. However, the adherence to HWE in our control group suggests that population stratification did not significantly bias our findings. Future research utilizing ancestry informative markers would be valuable to further refine these risk profiles in specific sub-populations.

Future studies integrating functional assays, including transcriptomic evaluations in matched tumor tissues, and independent population-based replication will be essential to validate these findings and to better define the biological impact of these regulatory variants within the oncogenic context.

## 5. Conclusions

This study provides evidence that regulatory variants within *KRAS* are associated with BC susceptibility in Mexican women, with consistent associations observed for rs12228277, rs1137196, rs8720 and rs12245 after multiple testing correction. The low linkage disequilibrium among these variants supports their independent effects, while multilocus analyses suggest that their combinatorial patterns may influence disease risk. Although associations with clinicopathological features were limited at the single-variant level, specific genotypic combinations showed nominal associations with tumor-related characteristics, highlighting the potential relevance of combinatorial regulatory variation. Functional annotations support a role for these variants in transcriptional and post-transcriptional regulation; however, further experimental validation and independent replication studies are required to confirm these findings and clarify their biological and clinical implications.

## Figures and Tables

**Table 1:** Clinical and epidemiological characteristics of the study population.

Variable	BC (n = 650)	Controls (n = 492)	*p*
Age (mean ± SD)	53.26 ± 11.31	53.84 ± 11.64	0.412
Alcohol use (%)	16	33	0.0001
Tobacco use (%)	22	31	0.001
Menstrual status			
Postmenopausal (%)	64	–	–
Premenopausal (%)	36	–	–
Body mass index			
Normal weight (%)	30	–	–
Overweight (%)	35	–	–
Obesity (%)	35	–	–
Family history			
Breast cancer (%)	20	–	–
Other type (%)	8	–	–
No history (%)	72	–	–
Clinicopathological history			
T2DM (%)	15	–	–
Hypertension (%)	6	–	–
Benign breast diseases (%)	8	–	–
Uterine fibrosis (%)	26	–	–
No history (%)	45	–	–
Pregnancies			
≤4 (%)	69	–	–
>4 (%)	18	–	–
No pregnancies (%)	13	–	–
Abortions			
Has had abortions (%)	20	–	–
No abortions (%)	80	–	–
Breastfeeding (months)			
≤6 (%)	35	–	–
>6 (%)	48	–	–
No (%)	17	–	–
Tumor location			
Bilateral (%)	4	–	–
Unilateral (%)	96	–	–
Tumor classification			
Ductal (%)	91	–	–
Lobular (%)	7	–	–
Mixed (%)	2	–	–
Molecular tumor classification			
Luminal A (%)	39	–	–
Luminal B (%)	26	–	–
HER-2 positive (%)	15	–	–
Triple negative (%)	20	–	–
Positive lymph nodes (%)	59	–	–
Ki-67			
Ki-67 ≥ 20% (%)	59	–	–
Ki-67 < 20% (%)	41	–	–
Tumor stage			
I–II (%)	43	–	–
III–IV (%)	57	–	–
Metastasis (%)	23	–	–
Chemotherapy response			
No response (%)	22	–	–
Partial response (%)	11	–	–
No response due to recurrence (%)	24	–	–
Total response (%)	43	–	–
Chemotherapy toxicity			
Gastric (%)	50	–	–
Hematological (%)	30	–	–
Both (%)	10	–	–
Other (%)	10	–	–

BC, breast cancer cases; Controls, control group; SD, standard deviation; T2DM, type 2 diabetes mellitus; HER-2, human epidermal growth factor receptor 2; Ki-67, proliferation index; *p*-value adjusted by false discovery rate.

**Table 2:** Genotype and allele associations of *KRAS* variants with BC risk.

SNV	Model	Genotype	BC (n)	BC (%)	CG (n)	CG (%)	OR (95% CI)	*p*	pFDR
rs12228277 (BC n = 608/CG n = 489)	Codominant	*TT*	202	33	261	54	1 (ref.)	–	–
	Codominant	*TA*	297	49	192	39	2.00 (1.54–2.59)	<0.0001	<0.0001
	Codominant	*AA*	109	18	36	7	3.91 (2.57–5.95)	<0.0001	<0.0001
	Dominant	*TA + AA*	406	67	228	47	2.30 (1.80–2.94)	<0.0001	<0.0001
	Recessive	*AA*	109	18	36	7	2.75 (1.85–4.09)	<0.0001	<0.0001
	Alleles	*T*	701	0.5765	714	0.7301	1 (ref.)	–	–
	Alleles	*A*	515	0.4235	264	0.2699	1.99 (1.66–2.38)	<0.0001	<0.0001
rs1137196 (BC n = 428/CG n = 423)	Codominant	*TT*	134	31	144	34	1 (ref.)	–	–
	Codominant	*GT*	139	32	207	49	0.72 (0.52–0.99)	0.0513	0.0917
	Codominant	*GG*	155	36	72	17	2.31 (1.61–3.33)	<0.0001	<0.0001
	Dominant	*GT + GG*	294	69	279	66	1.13 (0.85–1.51)	0.4216	0.5547
	Recessive	*GG*	155	36	72	17	2.77 (2.01–3.82)	<0.0001	<0.0001
	Alleles	*T*	407	0.4755	495	0.5851	1 (ref.)	–	–
	Alleles	*G*	449	0.5245	351	0.4149	1.56 (1.28–1.88)	<0.0001	<0.0001
rs8720 (BC n = 377/CG n = 492)	Codominant	*TT*	83	22	137	28	1 (ref.)	–	–
	Codominant	*CT*	149	40	243	49	1.01 (0.72–1.42)	1.0000	1.0000
	Codominant	*CC*	145	38	112	23	2.14 (1.48–3.09)	<0.0001	0.0001
	Dominant	*CT + CC*	294	78	355	72	1.37 (1.00–1.87)	0.0588	0.0918
	Recessive	*CC*	145	38	112	23	2.12 (1.58–2.85)	<0.0001	<0.0001
	Alleles	*T*	315	0.4178	517	0.5254	1 (ref.)	–	–
	Alleles	*C*	439	0.5822	467	0.4746	1.54 (1.27–1.87)	<0.0001	<0.0001
rs12587 (BC n = 500/CG n = 442)	Codominant	*TT*	147	30	114	26	1 (ref.)	–	–
	Codominant	*GT*	226	45	218	49	0.80 (0.59–1.09)	0.1841	0.2707
	Codominant	*GG*	127	25	110	25	0.90 (0.63–1.28)	0.5886	0.7007
	Dominant	*GT + GG*	353	71	328	74	0.83 (0.63–1.11)	0.2432	0.3378
	Recessive	*GG*	127	25	110	25	1.03 (0.77–1.38)	0.8806	1.0000
	Alleles	*T*	520	0.5200	446	0.5045	1 (ref.)	–	–
	Alleles	*G*	480	0.4800	438	0.4955	0.94 (0.78–1.13)	0.5181	0.6476
rs12245 (BC n = 384/CG n = 243)	Codominant	*AA*	119	31	58	24	1 (ref.)	–	–
	Codominant	*AT*	141	37	125	51	0.55 (0.37–0.82)	0.0031	0.0065
	Codominant	*TT*	124	32	60	25	1.01 (0.65–1.56)	1.0000	1.0000
	Dominant	*AT + TT*	265	69	185	76	0.70 (0.48–1.01)	0.0563	0.0918
	Recessive	*TT*	124	32	60	25	1.45 (1.01–2.09)	0.0476	0.0915
	Alleles	*A*	379	0.4935	241	0.4959	1 (ref.)	–	–
	Alleles	*T*	389	0.5065	245	0.5041	1.01 (0.80–1.27)	0.9538	1.0000

BC, breast cancer cases; CG, control group; OR, odds ratio; CI, confidence interval; pFDR, *p*-value adjusted by false discovery rate.

**Table 3:** Nominal associations between SNVs and clinicopathological variables (*p* < 0.05).

SNV	Genotype	Clinicopathological Variable	OR (95% CI)	*p*-Value	pFDR
rs12587	*GT*	PR-positive	1.66 (1.08–2.55)	0.0218	0.3992
rs12587	*GT*	Ki-67 high	0.53 (0.32–0.87)	0.0126	0.3992
rs12228277	*AT*	Lymph node metastasis	0.68 (0.46–0.99)	0.0419	0.5758
rs12228277	*AT*	Luminal B	0.65 (0.45–0.93)	0.0187	0.3992
rs12228277	*AT*	PR-positive	0.60 (0.41–0.88)	0.0090	0.3992
rs12228277	*AA*	PR-positive	0.54 (0.33–0.87)	0.0118	0.3992
rs12245	*AT*	PR-positive	1.88 (1.12–3.17)	0.0174	0.3992
rs1137196	*GG*	Luminal B	1.64 (1.03–2.63)	0.0400	0.5758

SNV, single nucleotide variant; OR, odds ratio; CI, confidence interval; pFDR, *p*-value adjusted by false discovery rate; PR, progesterone receptor; Ki-67, cellular proliferation marker; Luminal B, molecular subtype of breast cancer characterized by higher proliferation and/or lower hormone receptor expression.

**Table 4:** Significant Genotypic Combinations Associated with BC Risk (FDR-adjusted).

Variant Pair	Reference (HomRef)	Combination	Cases (n)	Controls (n)	OR (95% CI)	*p*	pFDR
rs12228277_rs1137196	*TT_TT*	*AT_GG*	64	16	7.48 (3.72–15.07)	<0.0001	<0.0001
rs1137196_rs8720	*TT_TT*	*GG_CC*	50	20	4.79 (2.34–9.8)	<0.0001	0.0006
rs12228277_rs8720	*TT_TT*	*AT_CC*	43	46	3.02 (1.6–5.69)	0.00064	0.0106
rs8720_rs12587	*TT_TT*	*CC_GT*	63	37	2.98 (1.61–5.52)	0.00050	0.0106
rs12228277_rs12587	*TT_TT*	*AT_GG*	44	35	2.86 (1.53–5.33)	0.00114	0.0151
rs12228277_rs12587	*TT_TT*	*AT_TT*	43	36	2.72 (1.46–5.06)	0.00193	0.0213
rs12228277_rs12587	*TT_TT*	*AA_GT*	26	19	3.11 (1.49–6.5)	0.00292	0.0275
rs12228277_rs8720	*TT_TT*	*AA_TT*	13	10	4.2 (1.62–10.88)	0.00431	0.0356
rs12228277_rs1137196	*TT_TT*	*AA_GT*	24	15	2.99 (1.37–6.52)	0.00657	0.0434
rs1137196_rs12245	*TT_AA*	*TT_AT*	25	59	0.37 (0.19–0.74)	0.00602	0.0434

OR, odds ratio; CI, confidence interval; pFDR, *p*-value adjusted by false discovery rate.

**Table 5:** Nominal associations between multilocus genotypic combinations and clinical–pathological variables.

Clinical Variable	Variant Pair	Reference	Combination	OR (95% CI)	*p*-Value	pFDR
PR positive	rs12587_rs12245	*TT_AA*	*GT_AT*	5.01 (1.96–12.75)	0.0008	0.3444
Ki67 high	rs12587_rs12245	*TT_AA*	*GT_AT*	0.23 (0.09–0.60)	0.0030	0.6468
Lymph node mts	rs12228277_rs12245	*TT_AA*	*TT_AT*	4.36 (1.70–11.21)	0.0036	0.8205
PR positive	rs12228277_rs12587	*TT_TT*	*TT_GT*	3.73 (1.40–9.98)	0.0114	0.9599
Ki67 high	rs1137196_rs12587	*TT_TT*	*GT_GT*	0.34 (0.14–0.84)	0.0220	0.9599
ER positive	rs12228277_rs1137196	*TT_TT*	*AT_TT*	2.51 (1.12–5.64)	0.0307	1.0000
Luminal B	rs12228277_rs12245	*TT_AA*	*AT_AA*	0.32 (0.12–0.83)	0.0318	1.0000
PR_positive	rs1137196_rs12587	*TT_TT*	*GT_GT*	2.58 (1.12–5.92)	0.0359	1.0000
Luminal_B	rs12228277_rs12245	*TT_AA*	*AT_TT*	0.38 (0.15–0.95)	0.0418	1.0000

OR, odds ratio; CI, confidence interval; pFDR, *p*-value adjusted by false discovery rate.

**Table 6:** In silico functional annotation of the rs12228277 variant.

Section	Variable	Result/Description
RegulomeDB	Score	1f *
Functional evidence		TF binding + DNase (moderate evidence)
DNase I hypersensitivity		82 peaks in the region
ChIP-seq (identified TF)		ATF2
Chromatin states		Active enhancer; Weak enhancer; Genic enhancer; Strong transcription; Weak transcription
HaploReg v4.1	Regulatory state by tissue	Enhancer/DNase
Binding motifs altered		NF-κB, Mrg1::Hoxa9
DNase evidence		Accessible sites in multiple tissues
Functional annotation		Intronic regulatory region with enhancer activity
Overall interpretation	Functional implication	Evidence that rs12228277 participates in cis-regulation through modification of TF binding sites and chromatin accessibility

TF, transcription factor; DNase, DNase I hypersensitivity; ChIP-seq, chromatin immunoprecipitation sequencing; ATF2, activating transcription factor 2; NF-κB, nuclear factor kappa B; Mrg1::Hoxa9, composite transcription factor binding motif. * RegulomeDB score (1f) indicates that the variant is supported by caQTL data together with transcription factor (TF) binding and/or chromatin accessibility (DNase peak).

**Table 7:** eQTL results for *KRAS* in breast tissue.

SNV	NES	*p*-Value
rs12228277	0.0038	0.92
rs8720	−0.094	0.00069
rs12587	−0.093	0.00078
rs12245	−0.094	0.00069
rs1137196	No data *	No data *

NES: Normalized Effect Size. * No data on the GTEx Platform.

## Data Availability

Data and materials are available in the article.
